# Energy audit data for a resort island in the South China Sea

**DOI:** 10.1016/j.dib.2015.12.033

**Published:** 2015-12-31

**Authors:** M. Reyasudin Basir Khan, Razali Jidin, Jagadeesh Pasupuleti

**Affiliations:** College of Engineering, Universiti Tenaga Nasional, Jalan IKRAM – UNITEN, 43000 Kajang, Selangor, Malaysia

**Keywords:** Tioman, South China Sea, Load profile, Renewable energy, Resort Island, Energy audit

## Abstract

The data consists of actual generation-side auditing including the distribution of loads, seasonal load profiles, and types of loads as well as an analysis of local development planning of a resort island in the South China Sea. The data has been used to propose an optimal combination of hybrid renewable energy systems that able to mitigate the diesel fuel dependency on the island. The resort island selected is Tioman, as it represents the typical energy requirements of many resort islands in the South China Sea. The data presented are related to the research article “*Optimal Combination of Solar, Wind, Micro-Hydro and Diesel Systems based on Actual Seasonal Load Profiles for a Resort Island in the South China Sea*” [1].

## **Specifications Table**

**Table:** 

Subject area	*Energy*
More specific subject area	*Electrical Engineering*
Type of data	*Figure*
How data was acquired	*TNB Tioman*
*TNB Research*
*TNB Energy Services*
Data format	*Raw, filtered and analyzed data*
Experimental features	*Site survey*
*Power plant visits*
*Analysis of local development planning*
*Communication with local residents*
Data source location	*Tioman Island, South China Sea,*
2°47′47″N latitude and 104°10′24″E longitude
Data accessibility	*Data is provided in supplementary materials directly with this article*

## **Value of the data**

• This data can be used for other research fields that involve the usage of load profiles for a resort island in the South China Sea.
• The load profile data is valuable for determining potential installation of renewable energy systems for a resort island in the South China Sea.
• The data describes the seasonal load profiles on Tioman Island where other researchers may use this data as a benchmark on assessing the seasonal tourism impact on resort islands.
• Tioman island load data represents typical seasonal load profiles of many resort islands in the South China Sea. Hence, this data can also be used by researchers to assess other resort islands in South China Sea.

## 1 Data

The data given in the supplementary material comprises of energy audit data collected during site visits on Tioman Island in the South China Sea. The data includes Tioman Island background information, current electricity situation and load profiles.

## 2 Experimental design, materials and methods

The data was collected from a site survey that has been conducted on Tioman Island from 4th to 5th July 2013. The load profiles for 2009–2011 have been requested from power station on the island. Since the raw load profiles data are confidential, the data included in the supplementary material has been plotted in daily, monthly and seasonal basis. These data can be used to access the seasonal load profiles on many islands in the South China Sea due to seasonal tourism industry. Both diesel and mini-hydropower stations had been visited during the site survey which is guided by the power station technicians and staffs. Meanwhile, other data such as type of loads and typical islanders and tourist activities has been collected by communication with local residents on the island such as chalets, shops and café owners. The data presented in this article has been used for renewable energy assessments on Tioman Island [1].
